# Integrating reaction and analysis: investigation of higher-order reactions by cryogenic trapping

**DOI:** 10.3762/bjoc.9.214

**Published:** 2013-09-10

**Authors:** Skrollan Stockinger, Oliver Trapp

**Affiliations:** 1 Organisch-Chemisches Institut, Ruprecht-Karls-Universität Heidelberg, Im Neuenheimer Feld 270, 69120 Heidelberg, Germany

**Keywords:** benzyne, cryogenic CO_2_ trap, cycloaddition, Diels–Alder reaction, flow chemistry, gas chromatography, on-column reaction gas chromatography

## Abstract

A new approach for the investigation of a higher-order reaction by on-column reaction gas chromatography is presented. The reaction and the analytical separation are combined in a single experiment to investigate the Diels–Alder reaction of benzenediazonium-2-carboxylate as a benzyne precursor with various anthracene derivatives, i.e. anthracene, 9-bromoanthracene, 9-anthracenecarboxaldehyde and 9-anthracenemethanol. To overcome limitations of short reaction contact times at elevated temperatures a novel experimental setup was developed involving a cooling trap to achieve focusing and mixing of the reactants at a defined spot in a fused-silica capillary. This trap functions as a reactor within the separation column in the oven of a gas chromatograph. The reactants are sequentially injected to avoid undefined mixing in the injection port. An experimental protocol was developed with optimized injection intervals and cooling times to achieve sufficient conversions at short reaction times. Reaction products were rapidly identified by mass spectrometric detection. This new approach represents a practical procedure to investigate higher-order reactions at an analytical level and it simultaneously provides valuable information for the optimization of the reaction conditions.

## Introduction

**Figure 1:**
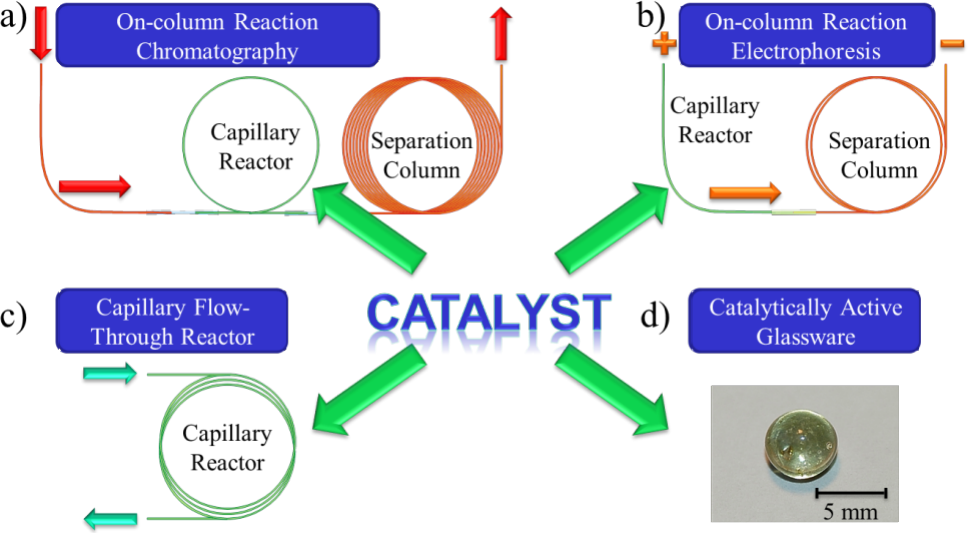
Overview of some capabilities of an immobilized or in an inert stationary phase dissolved catalyst. The use of reactive capillaries a) in on-column reaction GC, b) in on-column reaction capillary electrophoresis, c) as a capillary reactor to perform flow-through experiments, and d) to obtain catalytically active glassware.

The combination of synthesis and analysis in a single experiment offers many advantages. Time-consuming steps, i.e. work-up and separation of the reaction products, can be minimized, and direct analytical information about a reaction is obtained, including the formation of by-products and eventually even the reaction kinetics [1–4]. This information is important to design efficient flow-through experiments and to scale-up a reaction in a continuous process [5–9], which is often the first step for large-scale production. We contributed to this research field by developing on-column reaction chromatography, a technique where a catalytically active separation phase is used to perform (catalyzed) reactions and separations at the same time [10–17]. Here, the catalyst is immobilized or dissolved in an inert stationary phase and coated on the inside of fused-silica capillaries, which are then installed in a gas chromatograph (Figure 1a), capillary electrophoresis (Figure 1b) or liquid separation instrument (Figure 1c). In addition, the catalyst can be applied to the surface modification of glassware to obtain catalytically active bulk reactors, i.e. coated glass stir bars (Figure 1d).

The use of catalytically active fused-silica capillaries in analytical separation devices is a very powerful approach, because several reactions can be simultaneously performed by injecting a whole library of reactants at the same time and – most importantly – under exactly the same reaction conditions. Because of the separation of the reactant mixture, occurring prior to reaching the catalytically active section of the capillary, we do not observe competing reactions, so that the reaction kinetics can be investigated under comparable reaction conditions. So far, we investigated only first-order or pseudo-first-order reactions, where, for example, one reactant is used as a carrier gas, i.e., hydrogen in hydrogenation reactions or hydrogen peroxide as oxidant [18]. However, there is a great demand for investigating higher-order reactions, where several reactants are mixed and converted into a single product. We are currently developing an approach characterized by injecting a pulse of a slower migrating reactant, followed by a pulse of a faster migrating reactant. At a defined point the faster migrating reactant will “catch up” with the slower migrating reactant, interact, and react to the product. To cope with the limitations of applicable temperature ranges necessary to transport and separate the reactants and products, we developed a novel approach, which is discussed in the present contribution.

Here, we use a focusing technique to concentrate and stop separating several substances in a single column section to study higher-order reactions and to increase the conversion in on-column reaction gas chromatography (ocRGC). The major challenge in investigating multi-ordered reactions by ocRGC is the necessary contact of all reactants and catalysts involved in the reaction under the desired reaction conditions for a sufficient amount of time.

There are various techniques available to focus a substance within a gas chromatographic (GC) run at a defined point on a separation column. In general, focusing results in a decreased band width causing a concentration of the sample. We can take advantage of this effect in ocRGC, because the conversion is directly proportional to the concentration. Focusing is used for several reasons, namely to improve peak shapes, to increase the signal-to-noise ratio, and simply expand the detection limit of an analyst. In practice, focusing is achieved by cooling a short section of the separation column and reheating it after a certain amount of time. Frequently, cryogenic cooling traps are used. In most cases the cryogenic cooling device is installed directly in the GC oven and connected to the column at the desired column section. Reheating is performed by turning off the cooling device. Commercially available cryogenic cooling devices use compressed gas or high pressure liquids, such as CO_2_ or N_2_, to cool the column section. An interesting application of a cryogenic cooling trap is the longitudinally modulated cryogenic sampler (LMCS) used by Marriott et al. [19]. It is used to focus and concentrate samples between columns in a multidimensional gas chromatographic setup (GC×GC) [20].

**Scheme 1:**
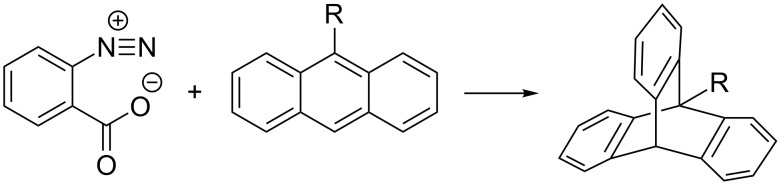
Diels–Alder reaction of benzenediazonium-2-carboxylate with anthracene-9-derivatives toward triptycene-9-derivatives.

To experimentally test our novel ocRGC setup, we chose the cycloaddition of benzyne with anthracene derivatives in a typical Diels–Alder-reaction (Scheme 1) as a model reaction.

This reaction seemed suitable because of the volatility of all reactants and products and the required reaction temperature, which is in an adequate range for GC measurements. Besides, the Diels–Alder reaction is a commonly used reaction and of broad interest. The used dienophile, benzyne, is highly reactive and must be generated in situ [21–24]. This can be achieved by chemical decomposition initiated by fluoride [25], lithium [22,26], an oxidizing agent [27] or by physical decomposition by radiation [28] or heating [29–31] of a precursor system. Here, we used benzenediazonium-2-carboxylate as a benzyne precursor, which thermally decomposes by elimination of nitrogen and carbon dioxide.

## Results and Discussion

**Figure 2:**
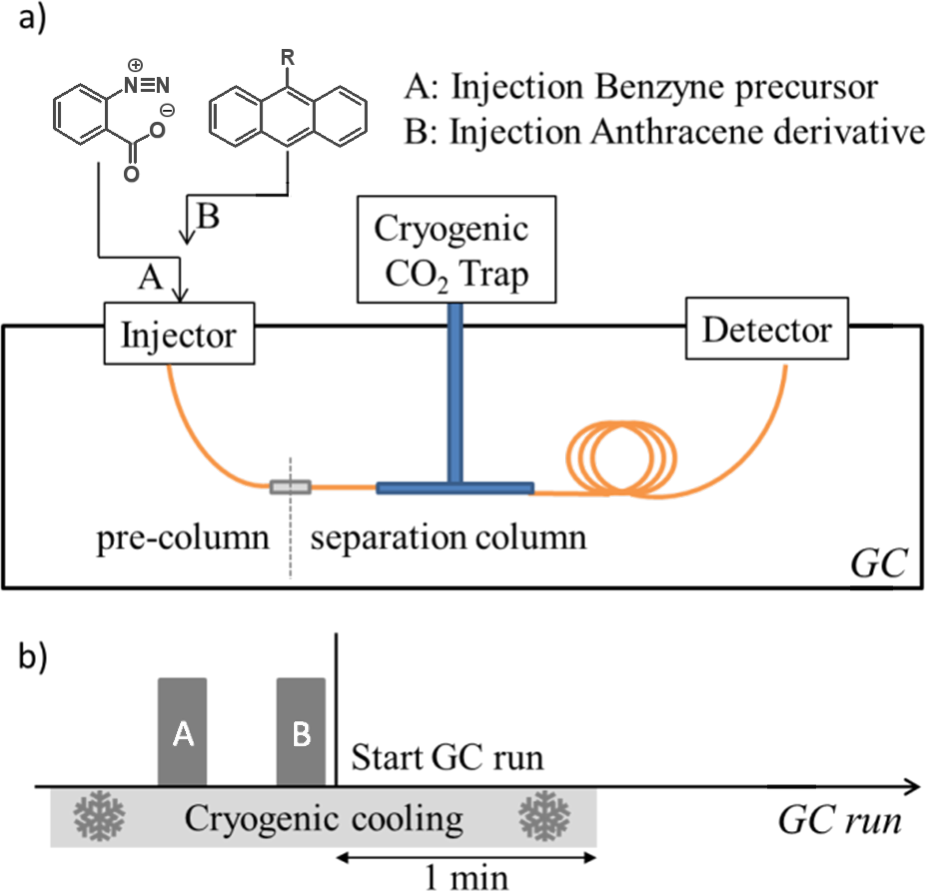
The experimental setup and a) the injection sequence protocol of the cryogenic ocRGC measurements b). Conditions: on-column cold injection, 60 °C for 5 min, then 6 °C/min to 180 °C, 120 kPa He; pre-column: fused silica, 10 cm, 250 nm film thickness, 250 µm i.d.; separation column: GE-SE30 (100% polydimethylsiloxane), 10 m, 500 nm film thickness, 250 µm i.d.

Initially, we chose to inject the heat-labile benzenediazonium-2-carboxylate after the anthracene derivative, because we expected that the thermally formed benzyne would be surrounded by the anthracene derivative, and an optimal conversion should occur. Surprisingly, the inverse injection order leads to a significant increase in yield, which might be explained by a certain lag time in the formation of benzyne. Therefore, the benzenediazonium-2-carboxylate was injected first, followed by the anthracene derivative by using two syringes to avoid any product formation by contamination. It can be assumed that the thermal activation of the benzyne formation is already initiated in the injector at elevated temperature. During this period, benzyne is collected in the capillary section covered by the cooling trap, leading to an enhanced reactivity when the anthracene derivative is injected into the cooling trap. Morgen et al. [32] published a procedure in which the GC injector is used as a chemical reactor to convert several reactants into a product. In contrast, our approach limits the intended reaction to a defined column section in the cooling trap. Figure 2 visualizes the experimental setup. First, the reactants were injected into a short pre-column, which is used to transfer them to the cryogenic cooling trap. The separation column is connected with the cooling trap, and the cooling zone extends for about 4 mm at the very beginning of this capillary. The cryogenic cooling is realized by using a cryogenic CO_2_ cold trap system directly installed in the GC oven. The cooling is started before the injection of the first reactant, so that all reactants are focused in this section. All reactants are collected in the cooling trap by this experimental setup. Thus, an unintended reaction occurring in the injector prior to the reaction taking place in the capillary section cooled by the cooling trap is avoided. After all reactants are injected, the GC run is started. It was found that the optimal cryogenic cooling time has to be extended by 1.0 min measured from the start of the measurements. The application of longer or shorter cooling times resulted in lower conversions, which might be explained by a spatial separation in case of cooling temperatures for longer cooling times and by the incomplete condensation of the reactants at the defined cooling section for too short cooling times, respectively. A fused silica column coated with chemically inert polydimethylsiloxane (GE-SE30) was used as a separation column to avoid any side reactions of the highly reactive benzyne with the stationary phase. It has to be pointed out that our approach is not limited to the use of neat polydimethylsiloxane, but other non-reactive stationary phases may be used, too. One of the advantages of the described experimental setup is the possibility to continuously tune the polarity and the properties of the stationary phase thereby optimizing the conditions for a particular reaction.

**Figure 3:**
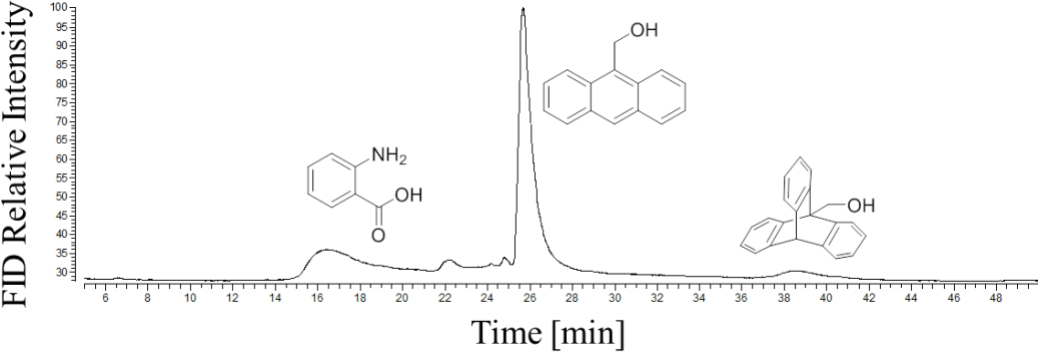
Experimental ocRGC chromatogram of 9-anthracenemethanol with benzenediazonium-2-carboxylate. Conditions: on-column cold injection, 60 °C for 5 min, then 6 °C/ min to 180 °C, 120 kPa He; pre-column: fused silica, 10 cm, 250 nm film thickness, 250 µm i.d.; separation column: GE-SE30 (100% polydimethylsiloxane), 10 m, 500 nm film thickness, 250 µm i.d.

A typical chromatogram of the on-column Diels–Alder reaction with 9-anthracenemethanol is shown in Figure 3. Anthranilic acid was identified as the decomposition product of benzenediazonium-2-carboxylate in these ocRGC experiments. It is already formed by the sole injection of benzenediazonium-2-carboxylate. An overview of the obtained yields of the formed triptycene-9-derivatives is given in Table 1. The yields are reproducible between 1.4 and 6% as expected for the very short reaction times during the heating step. All reactions were also performed by a regular synthesis in test tubes in order to compare the results with those of the ocRGC measurements (see Table 1).

**Table 1:** Yield of triptycene-9-derivatives.^a^

R =	Synthesis in a flask				ocRGC^b^			
	*t*	*T*	Yield	*k* [s^−1^]	*t*	*T*	Yield	*k* [s^−1^]

H	24 h	rt	11%	1.4 × 10^−6^	~1s	60 °C	^c^	
	3 h	80 °C	31%	3.4 × 10^−5^				
Br	24 h	rt	7%	8.4 × 10^−7^	~1s	60 °C	1.8%	1.8 × 10^−2^
	3 h	80 °C	28%	3.0 × 10^−5^				
CHO	24 h	rt	4%	4.7 × 10^−7^	~1s	60 °C	1.4%	1.4 × 10^−2^
	3 h	80 °C	13%	1.3 × 10^−5^				
CH_2_OH	24 h	rt	16%	2.2 × 10^−6^	~1s	60 °C	6.0%	6.2 × 10^−2^
	3 h	80 °C	44%	5.4 × 10^−5^				

^a^100% coverage of the carbon-corrected FID area of anthracene-9-yl derivative and the associated triptycene-9-yl derivative. ^b^Average values of at least three measurements are shown. The average standard deviation of the values does not exceed ±1%. ^c^A detectable but infinitesimal small amount of transformation.

The test tube system gives only moderate yields, even if the reaction temperature is raised to 80 °C. According to Stiles et al. the reaction of anthracene and benzenediazonium-2-carboxylate in refluxing benzene yields 30% triptycene with a reaction time of 64 hours [30]. Our measurements revealed that this yield could already be achieved after 3 hours. This could also be experimentally proven by the test-tube experiment. In both systems, test tube and ocRGC, the best yields were achieved with 9-anthracenemethanol as a diene component. For the test-tube system a yield of 16% after 24 hours at room temperature and 44% after 3 hours at 80 °C was found. The reaction time for the on-column reaction is considerably smaller, because an effective contact time of less than one second must be assumed. Consequently, the measured yield of 6% of 9-triptycenemethanol for the on-column measurement can be considered as an impressing result. Unfortunately, all other anthracene derivatives did not exceed a yield of 2% with the ocRGC method. For the test-tube experiments the yield of 9-anthracenemethanol is also higher than those of the other derivatives, but the differences are samller compared to the on-column system. Surprisingly, the substituents of the anthracene derivatives show a more pronounced effect on the conversion compared to the classical synthesis in a flask, which might be explained by differences in the relative migration velocities of the dienes. We calculated reaction rate constants from our experiments (*k*, Table 1), which clearly indicate that a large surface area substantially improves the rate of conversion. We observed an acceleration of a magnitude of approximately 1000 under these conditions. Nevertheless, both systems show the same tendency of an electronic influence of the substituents on the reactivity. Thus, the reactivity order is not affected by our novel approach. In contrast to a classical reaction screening, our on-column gas chromatographic setup can be used to determine this reactivity order within a few hours, including the complete separation and characterization of all reactants, products and side-products and requiring only minute amounts of the reactants. Consequently, the method facilitates a complete screening of large substance libraries within a few days. Additionally, all detectable multi-order reactions may be investigated without any modification of the illustrated setup. Despite the advantage of an enrichment of the in-line sample concentration by the cryogenic CO_2_ setup, reactions can be studied in greater detail. Individual reaction steps can be slowed down by cooling only locally at different positions of a catalytic column, and several substances can be pre-focused before the actual reaction occurs to allow for an easier investigation of the reaction process. In summary, the presented ocRGC setup with a cryogenic CO_2_ cooling trap is a fast and accurate screening method for reactivity investigations of multi-order reactions.

## Experimental

Benzenediazonium-2-carboxylate was synthesized according to a procedure described in [33–34]. Anthracene, 9-bromoanthracene, 9-anthracenecarboxaldehyde and 9-anthracenemethanol were purchased from Aldrich Chemical, Acros or ABCR and used without further purification. As GC–MS a Trace GC Ultra combined with an ISQ Single Quadrupole mass spectrometer, both from Thermo Scientific, were used. For cryofocusing a Cryogenic CO_2_ Cold Trap System by SGE Analytical Science was installed, according to the experimental setup outlined in Figure 2. For cryofocusing CO_2_ was used as a cooling liquid [35]. For the measurement of the reference substances 50 mg of benzenediazonium-2-carboxylate was mixed with 0.8 equiv anthracene or the anthracene derivative in 2 mL 1,2-dichloroethane or dichloromethane (DCM) and stirred under the conditions given in Table 1. After filtration and evaporation the reaction mixture was dissolved in pentane and washed with water. The diluted pentane solution was analyzed by GC–MS. For the ocRGC analysis separate DCM solutions of anthracene or anthracene derivative and benzenediazonium-2-carboxylate were prepared, both containing 1 mg per mL. The injection was performed by using two different on-column syringes from SGE (experimental setup is outlined in Figure 2) following the general on-column cold injection techniques with a secondary N_2_-cooling time of 2 min. For analysis the FID signal was used and carbon-corrected.
